# Differential Growth Inhibitory Effects of Highly Oxygenated Guaianolides Isolated from the Middle Eastern Indigenous Plant *Achillea falcata* in HCT-116 Colorectal Cancer Cells

**DOI:** 10.3390/molecules18078275

**Published:** 2013-07-15

**Authors:** Rita Tohme, Lamis Al Aaraj, Tarek Ghaddar, Hala Gali-Muhtasib, Najat A. Saliba, Nadine Darwiche

**Affiliations:** 1 AUB Nature Conservation Center, American University of Beirut, Beirut, P.O. Box 11-0236, Lebanon; E-Mails: rgt07@aub.edu.lb (R.T.); la70@aub.edu.lb (L.A.A.); tg02@aub.edu.lb (T.G.); amro@aub.edu.lb (H.G.-M.); 2 Department of Biology, American University of Beirut, Beirut, P.O. Box 11-0236, Lebanon; 3 Department of Chemistry, American University of Beirut, Beirut, P.O. Box 11-0236, Lebanon; 4 Department of Biochemistry and Molecular Genetics, American University of Beirut, Beirut, P.O. Box 11-0236, Lebanon

**Keywords:** *Achillea falcata*, sesquiterpene lactones, guaianolides, colorectal cancer cells, bioassay-guided fractionation, structure-activity relationship

## Abstract

Medicinal plants play a crucial role in traditional medicine and in the maintenance of human health worldwide. Sesquiterpene lactones represent an interesting group of plant-derived compounds that are currently being tested as lead drugs in cancer clinical trials. *Achillea falcata* is a medicinal plant indigenous to the Middle Eastern region and belongs to the Asteraceae family, which is known to be rich in sesquiterpene lactones. We subjected *Achillea falcata* extracts to bioassay-guided fractionation against the growth of HCT-116 colorectal cancer cells and identified four secotanapartholides, namely 3-β-methoxy- isosecotanapartholide (**1**), isosecotanapartholide (**2**), tanaphallin (**3**), and 8-hydroxy-3-methoxyisosecotanapartholide (**4**). Three highly oxygenated guaianolides were isolated for the first time from *Achillea falcata*, namely rupin A (**5**), chrysartemin B (**6**), and 1β, 2β-epoxy- 3β,4α,10α-trihydroxyguaian- 6α,12-olide (**7**). These sesquiterpene lactones showed no or minor cytotoxicity while exhibiting promising anticancer effects against HCT-116 cells. Further structure-activity relationship studies related the bioactivity of the tested compounds to their skeleton, their lipophilicity, and to the type of functional groups neighboring the main alkylating center of the molecule.

## 1. Introduction

Traditionally, medicinal plants have been used to treat all kinds of human diseases [1]. The main sources of bioactivity in medicinal plants are the plant secondary metabolites they produce [2]. Secondary metabolites are low molecular weight compounds and encompass different types of molecules, namely sesquiterpene lactones [2,3]. The latter are categorized into the following major closed ring structures groups: germacranolides, eudesmanolides, eremophilanolides, guaianolides, pseudoguaianolides, and hypocretenolides [3]. However, some also bear open ring structures such as isosecotanapartholides [4].

Sesquiterpene lactones have enhanced inflammatory and anticancer properties, which render them promising anticancer drugs [5,6]. Parthenolide, artemisinin, and thapsigargin are examples of such compounds and have already reached cancer clinical trials [3]. The main reactive moiety in sesquiterpene lactones is their exocyclic α-methylene-γ-lactone ring, which forms stable adducts with biological nucleophiles through a Michael-type addition, and therefore, is the main drive behind the bioactivity of these compounds [7]. The biological activity of these molecules has been mainly attributed to the number of alkylating centers, the geometry of the molecule, as well as to their lipophilicity [3].

Previously, the medicinal plant indigenous to the Middle Eastern region, *Achillea falcata*, which belongs to the Asteraceae family, was shown to be a rich source of sesquiterpene lactones [4]. Four different sesquiterpene lactones were isolated from *Achillea falcata*, namely 3-β-methoxy-isosecotanapartholide (**1**), isosecotanapartholide (**2**), tanaphillin (**3**), and 8-hydroxy-3-methoxy-isosecotanapartholide (**4**). These secotanapartholides differentially decrease the growth of the HaCaT keratinocyte cell line [4]. The effect of compound **1** on the NF-κB and AP-1 signaling pathways was investigated and compound (**1**) was shown to modulate the activity of these transcription factors in cellular transformation and tumorigenesis [8]. Compound **2** was shown to induce apoptosis in HCT-116 colorectal cancer cells when combined with another sesquiterpene lactone, salograviolide A, through a caspase-independent mechanism [9].

Considering the increase in interest in the anticancer properties of sesquiterpene lactones [3], we investigated the presence of other bioactive ones in *Achillea falcata*. Therefore, the bioassay-guided fractionation of extracts from the aerial parts of *Achillea falcata* was performed against the growth of the human colorectal cancer cell line HCT-116. Indeed, colorectal cancer is one of the most prevalent cancers in the World and in the Middle East [10,11,12] and HCT-116 is one of the most widely used and characterized *in vitro* model of colorectal cancer [13].

## 2. Results and Discussion

### 2.1. *Achillea falcata* Extract Rich in Sesquiterpene Lactones Decreases the Growth of HCT-116 Colorectal Cancer Cells

Following the acid-base extraction described in the Experimental section, a fraction rich in sesquiterpene lactones was obtained and was labeled “I.2”. The I.2 fraction was tested against a panel of cancer cell lines that are prototype of some human cancers such as colorectal (HCT-116), epidermal squamous cell carcinoma (HaCaT-II4), promyelocytic leukemia (HL-60), and T-lymphoma (Jurkat) cells using an MTT-based cell growth assay. The I.2 extract caused a dose-dependent decrease in viability (results not shown), which was more pronounced in leukemia and lymphoma cell lines as the half maximal inhibitory concentration (IC_50_) values were approximately 4 µg/mL, and at least three fold lower than those of tested solid tumors (Table 1)*.*

**Table 1:** IC_50_ values of *Achillea falcata *I.2 fractions for inhibition of human cancer cell growth at 24 h. Results represent the averages ± SE of three independent experiments done in triplicate wells.

	HCT-116	HaCaT II4	HL-60	Jurkat
**Average IC_50_** **I.2 Extract (μg/mL) ± SE**	21.0 ± 2.08	11.7 ± 0.54	4.6 ± 0.34	4.0 ± 0.54

The bioassay-guided fractionation of *Achillea falcata* was further pursued on the growth of HCT-116 cells as colorectal cancer is a major public health problem in the World as well as in the Middle East. HCT-116 cells were previously shown to be sensitive to the sesquiterpene lactone salograviolide A, isolated from *Centaurea ainetensis,* and the β-isomer of isosecotanapartholide (**2**), which inhibited the growth of HCT-116 with IC_50_ values of 8 and 10 μg/mL, respectively, at 24 h [9].

### 2.2. The Bioactive I.2 Sub-Fractions are Rich in Sesquiterpene Lactones

**Figure 1:**
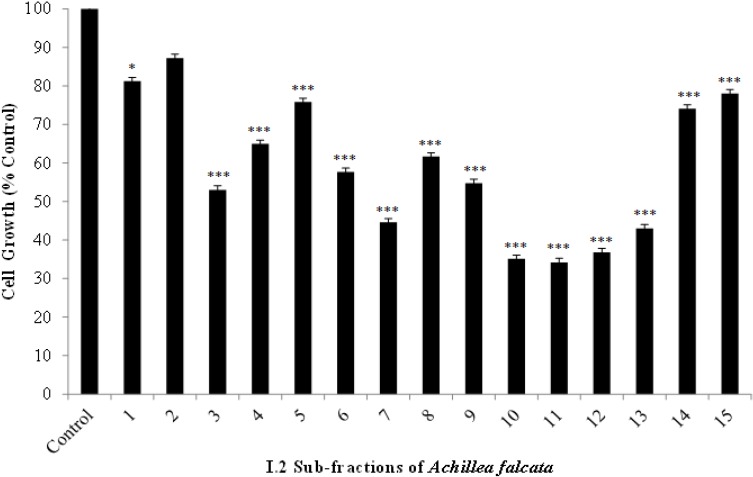
Effects of the I.2 sub-fractions of *Achillea falcata* extract on the viability of HCT-116 cells. Cells were plated in 96-well plates at a density of 1x10^5^ cells/mL and treated at 40%–50% confluency with vehicle ethanol or a concentration of 25 μg/mL up to 24 h. Results are percentage of control and plotted as averages of quadruplet wells (±SD). Results are representative of two independent experiments. Statistical significance from control is indicated as * when *p *< 0.05, and as *** when *p* < 0.001 for I.2 subfractions.

The bioactive extract I.2 was further fractionated using solid-liquid chromatography and a total of 15 different sub-fractions were collected and tested against HCT-116 cells (Figure 1). The effect of the different sub-fractions on cell growth at 24 h was determined at a concentration of 25 μg/mL, which is close to the calculated IC_50_ value (Table 1). Fractions 10–13 were the most potent and significantly decreased the growth of HCT-116 cells up to 60%–70% of control. Nuclear magnetic resonance (NMR) spectra of these fractions showed two doublets typically appearing in the 5.50–6.50 region (with *J* = 1–4 Hz), characteristic fingerprints of the α-methylene-γ-lactone hydrogens of the sesquiterpene lactones [14].

**Figure 2:**
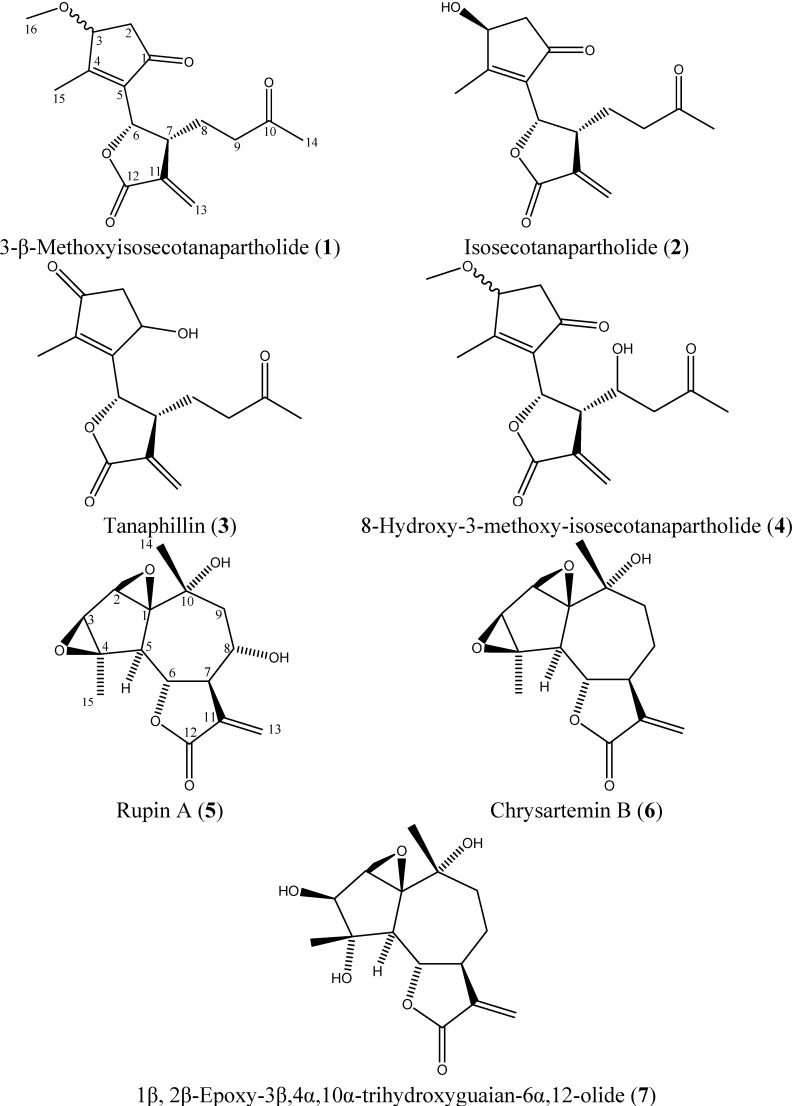
Chemical structures of sesquiterpene lactones isolated from *Achillea falcata* **1**–**7**.

Further purification of the most bioactive sub-fractions 10–13 revealed the presence of seven different sesquiterpene lactones (Figure 2). In addition to 3-β-methoxyisosecotanapartholide (**1**), an α/β mixture of isosecotanapartholide (**2**), and tanaphillin (**3**), three compounds were isolated for the first time from *Achillea falcata*, namely rupin A (**5**, 4 mg), chrysartemin B (**6**, 6 mg), and 1β, 2β-epoxy-3β,4α,10α-trihydroxyguaian- 6α,12-olide (**7**, 6.5 mg). The presence of other sesquiterpene lactones, including 8-hydroxy-3-methoxy-isosecotanapartholide (**4**), was also detected; however these compounds were present in very low amounts and were not isolated. 

The spectroscopic data of compounds **5**–**7** were identical to those reported in the literature [15,16,17,18,19,20]. Compound **5** has already been purified from several Asteraceae species such as *Artemisa tripartita* [15], *Artemisia ludoviciana* [21], * Ajania fastigiata* [22], *Achillea biebersteinii* [23], *Achillea crithmifolia* [24], and *Achillea setacea* [25]. Among these plants, the plant extract from *Achillea biebersteinii* was the only one previously tested for its cytotoxic activity against several cancer cell lines [26]. Compound **6** was previously isolated from *Artemisia aschurbajevii* [27], *Artemisia ludoviciana* [28], *Artemisia ludoviciana* spp. *mexicana* [29], *Chrysanthemum parthenium* [20], *Chrysanthemum morifolium* [30], *Achillea ligustica* [31], *Achillea santolina* [32], and *Handelia trichophylla* [33]. Compound **7** was isolated from *Pentzia albida* [16], *Tanacetum cilicium* [19], *Artemisia anomala* [34], *Artemisia deserti* [35], and *Ajania fruticulosa* [36]. The methanolic extract of *Artemisia anomala* was found to possess antibacterial and antioxidant properties [37]. Furthermore, the isolated chlorine derivative of (**7**) from *Artemisia anomala* showed cytotoxic activity against numerous human cancer cell lines [38].

### 2.3. Isolated Sesquiterpene Lactones from *Achillea falcata* Exhibit Dose-Dependent Growth Inhibitory Effects on HCT-116 Cancer Cells

**Figure 3:**
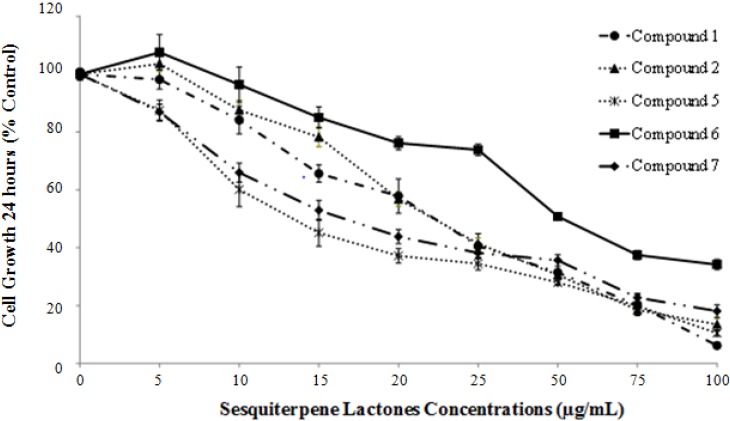
Effects of sesquiterpene lactones (**1**), (**2**), and (**5**–**7**) from *Achillea falcata* on the growth of HCT-116 cells. Cells were plated in 96 well plates at a density of 1×10^5^ cells/mL. At 40%–50% confluency, cells were treated for 24 h with different concentrations of sesquiterpene lactones (**1**), (**2**), and (**5**–**7**) up to 100 μg/mL. We used an MTT assay as explained in experimental sections. Results represent the averages (±SE) of at least three independent experiments done in triplicate wells.

The isolated compounds **1**, **2**, **5**–**7** were tested for their cytotoxic and growth inhibitory effects on HCT-116 cells. Compounds **3** and **4** were not tested as **3** is highly unstable and isomerized into **2** while **4** was not successfully isolated due to its presence in minute amounts. First, cytotoxicity was determined at 6 h as measured by lactate dehydrogenase (LDH) release and expressed as percentage of control-treated cells for each group (Table 2). None of the tested sesquiterpene lactones exceeded 38% cytotoxicity at 6 h treatment with concentrations ranging from 5 to 100 µg/mL (Table 2). Sesquiterpene lactones **1**, **2**, and **6** did not possess any detectable cytotoxicity up to 100 µg/mL. Triton-X 100, with an IC_50_ lower than 1 × 10^−1^ µg/mL at 6 h, was used as a positive control (Table 3). These results reflect the non-acute mode of action of the tested compounds against HCT-116 cells. Subsequently, the effect of **1**, **2**, and **5**–**7 **was determined on cell growth at concentrations ranging from 5 to 100 µg/mL using the MTT assay which measures the ability of cells to metabolize tetrazolium salt (MTT) into blue formazan (Figure 3). Treatment with sesquiterpene lactones **1**, **2**, **5**–**7** caused a dose-dependent growth inhibition of HCT-116 cells. Compound **5** was the most active with an IC_50_ of 15.2 ± 0.93 µg/mL while (**6**) was the least active with an IC_50_ of 67.0 ± 3.99 µg/mL (Table 4). 

In this study, the growth inhibitory effects of compounds **1**, **2**, **5**–**7** were tested against HCT-116 cells. As stated previously, the potency of the tested sesquiterpene lactones is primarily attributed to the characteristic alkylating center of sesquiterpene lactones, the α-methylene-γ-lactone functional group, which targets and inhibits the activity of several functional proteins [7]. However, the observed differential growth inhibitory effects may be also attributed to the difference in the skeleton and/or the attached functional groups [3] (Table 4).

**Table 2:** Cytotoxicities at 6 h of isolated sesquiterpene lactones **1**, **2**, and **5**–**7** on HCT-116 cells. Results are percentage of control and represent the averages (±SE) of three independent experiments done in triplicate wells.

		Sesquiterpene Lactone Concentrations (μg/mL)								
Compounds		0	5	10	15	20	25	50	75	100
**1**		0 ± 0.5	0 ± 0.9	0 ± 0.7	0 ± 0.7	0 ± 1.9	0 ± 0.8	0 ± 1.0	3 ± 2.7	0 ± 2.1
**2**		0 ± 0.5	0 ± 0.5	0 ± 0.3	0 ± 2.1	0 ± 0.3	0 ± 0.5	0 ± 1.7	0 ± 0.6	0 ± 1.3
**5**		0 ± 0.5	0 ± 0.2	0 ± 0.3	0 ± 0.8	0 ± 0.7	2 ± 0.3	16 ± 0.7	16 ± 0.4	38 ± 0.2
**6**		0 ± 0.6	0 ± 1.4	0 ± 1.7	0 ± 2.2	0 ± 2.5	0 ± 3.2	0 ± 2.4	0 ± 3.0	0 ± 3.8
**7**		0 ± 0.6	0 ± 2.0	2 ± 1.4	1 ± 1.3	3 ± 1.0	2 ± 0.8	18 ± 4.0	24 ± 1.7	18 ± 4.2

**Table 3:** Cytotoxicity at 6 h of Triton-X 100 on HCT-116 cells.

Concentrations (μg/mL)								
			**0**	**0.1**	**1**	**10**	**100**	**1000**
**Positive Control **			Cytotoxicity at 6 h (% Control) ± SE					
**Triton X**			0 ± 1.0	1 ± 1.9	0 ± 0.9	3 ± 0.5	75 ± 3.5	74 ± 2.5

**Table 4:** IC_50_ values at 24 h of sesquiterpene lactones **1**, **2**, and **5**–**7** for inhibition of HCT-116 cell growth. Results are tabulated as averages of at least three independent experiments ± SE.

Compounds	Average IC_50_ (µg/mL)	±SE
Compound **1**	25.6	1.52
Compound **2**	29.4	1.07
Compound **5**	15.2	0.93
Compound **6**	67.0	3.99
Compound **7**	19.3	1.02

When comparing the activities of the two secotanapartholides **1** and **2**, compound **1** shows higher activity against the HCT-116 cells. Higher potency was attributed to the effect of the methoxy (CH_3_O) group substitution at C-3 on the lipophilicity of the molecule, and hence, its ability to cross the plasma membrane [4].

In reference to the difference in basic skeleton, it is noticed that the open guaianolides, secotanapartholides **1** and **2**, are significantly less active than the closed guaianolides **5** and **7** at equivalent concentrations up to 25 µg/mL. This confirms that one of the key determinants of activity is the 5-7-5 ring system, as suggested based on docking studies of guaianolides [39,40] and in experimental structure-activity relationship studies [41,42,43]. Moreover, it was shown that the acid catalyzed intramolecular cyclization of the germacranolide parthenolide into a guaianolide enhanced the anti-inflammatory potential of that molecule [42,43]. Other factors such as the hydrophobic/hydrophilic substituents at C-3, C-4, and C-8 positions play a complementary role to the basic structure and to the α-methylene-γ-lactone functional group in enhancing the activity of the molecule. 

In fact, the role played by the substituents at the different positions was best illustrated when the potency of the three 5-7-5 ring guaianolide structures were compared. Previous studies highlight the direct correlation between the partition coefficient of a molecule and its inhibitory effect [44], which led to the synthesis of water-soluble derivatives of drugs such as parthenolide and micheliolide [45]. However, in this study, while compound **5** was the most potent, it was shown to have the lowest solubility in water, followed by **6** and **7** (data not shown). Furthermore, although **6** has the lowest number of hydroxyl groups, and therefore, greater lipophilicity when compared to compounds **5 **and **7**, it was shown to be the least active. Hence, the relative solubility of the molecules and their lipophilicity did not correlate with their inhibitory activities. The differential effect of compounds **5**–**7** was thus primarily attributed to the identity and the positions of the substituents present on their basic skeletons.

Compound **5** has an enhanced potency over compound **6** as the extra hydroxyl group at position C-8 next to the α-methylene-γ-lactone moiety significantly increased the activity of this molecule. The presence of hydroxyl groups at position C-8 has been previously shown to affect the activity of several sesquiterpene lactones [46,47,48,49]. Furthermore, the opening of the epoxide ring at C-3 and C-4 into diols enhanced the activity of **7** over **6**. Neighboring hydroxyl groups to the molecule’s main alkylating center have been reported to enhance the rate of cysteine addition by presumably facilitating the addition of the sulfur anion RS^−^, or the proton transfer at an intermediate stage in the Michael-type addition [46]. This is best illustrated in compounds **5** and **7**, where the presence of hydroxyl groups at positions C-8 and C-4, respectively, had a similar effect on the potency of the molecule. One could also suggest that despite the fact that oxygen atoms in epoxide and hydroxyl functional groups can both participate in intermolecular hydrogen bonding with amino acid residues adjacent to the reactive center of the target protein [46], the greater strength of hydrogen bonding, and/or the conformational flexibility of a hydroxyl group, might be reducing the steric hindrance of the molecule in a way to enhance its anti-proliferative effect.

In this study using the HCT-116 cell line model, careful analysis of binding site interactions have revealed, in addition to the α-methylene-γ-lactone moiety, three major structural features important for the activity within this series of compounds, namely the (i) 5-7-5 ring system, (ii) hydroxyl bonding at a site neighboring the molecule’s main alkylating center (C-4 and C-8 in this case) and (iii) the presence of a hydrophobic substituent at the C-3 position as confirmed by other studies [4,39,40]. Hence, the replacement of the hydroxyl group at C-3 by a hydrophobic group in compound **7** may increase the biological activity of this molecule even further, rendering it a promising potent drug for future studies.

## 3. Experimental

### 3.1. General

All deuterated and non-deuterated solvents were purchased from Acros Organics, Geel, Belgium. The silica cartridges and silica gel were obtained from Grace, Alltech Associates, Inc., Deerfield IL, USA, and from Fluka, Sigma-Aldrich, St. Louis MO, USA, respectively. Dulbecco’s modified Eagle’s medium (DMEM) nutrient and Roswell Park Memorial Institute 1640 medium nutrient were obtained from Bio Whittaker, Cambrex Co., East Rutherford, NJ, USA and phosphate-buffered saline (PBS), heat inactivated fetal bovine serum (FBS), glucose, sodium pyruvate, trypsin, kanamycin, penicillin-streptomycin, trypan blue dye, and non-essential amino acids were obtained from Gibco-BRL Life Technologies, Carlsbad, CA, USA.

### 3.2. Harvesting and Processing of Achillea falcata

The aerial parts of *Achillea falcata* were harvested from the mountainous areas of Lebanon at an altitude of 1,450–1,600 m, in the flowering season of the plant in July 2011, and processed for extraction. The plant shoots were left to air dry for a week in the shade inside the Agricultural Research and Education Facility of the American University of Beirut, Lebanon. When fully dried, the plant material was grinded and sealed in vacuum bags. Moisture content was determined by pre-weighting and post-weighting 20 g of dried plant material after incubation in the oven at 55 °C every 15 min until weight stabilization. This experiment was repeated three times and average water content for harvested *Achillea falcata* was found to be around 10% of the total weight (data not shown). 

### 3.3. Extraction and Purification of Sesquiterpene Lactones

Extracts from the aerial parts of *Achillea falcata* (200 g) were prepared as previously described [50,51]. Briefly, the aerial parts were soaked, separately, in 2 L methanol for 16 h at room temperature. The crude methanolic extracts “I” were concentrated to 1/10 of their volumes and acidified to pH = 2 with a sulfuric acid solution. Liquid-liquid extraction using a mixture of chloroform (CHCl_3_):water (2:1 v/v) followed and the organic layer “I.2” was collected and evaporated under reduced pressure at 40 °C to give 6.82 g. I.2 was assayed for its growth inhibitory activities against HCT-116 human colorectal cancer cell line. I.2 was applied to a liquid column chromatography (silica gel 0.035–0.075 mm, 60 Å, 1 kg) and fractionated using a gradient elution of CHCl_3_:acetone (9:1), CHCl_3_:acetone (2:1), acetone, and methanol. Fifteen sub-fractions were collected and were concentrated *in vacuo* at 40 °C. The residues were weighed, dissolved in ethanol, and assayed for their growth inhibitory activities against HCT-116 cells. The fractions containing sesquiterpene lactones were purified to isolate the bioactive molecules using a preparative high performance liquid chromatography (HPLC) Gilson GX Prep coupled with ultraviolet detection (UV/VIS-156). The column compartment is a Reprosil 100 C18 column (150 x 50 mm i.d.; 10 μm). The pre-column is a Reprosil 100 C18 column (50 × 50 mm i.d.; 10 μm). The injection volume was 4000 µL for all samples. The mobile phase consisted of an isocratic elution of acetonitrile and water (30:70) for 100 min. The flow rate was set to 12 mL/min and the wavelengths of the ultraviolet detector at 205 and 220 nm at room temperature. 

### 3.4. Structure Elucidation

The structure of the bioactive sesquiterpene lactones was elucidated using a Nicolet AVATAR 360 Fourier Transform Infrared (FTIR) spectrometer equipped with a KBr pellet cell holder. Spectra were collected by averaging 128 scans at wave numbers ranging from 750 to 4,000 cm^−1^ at a resolution of 1 cm^−1^. A Bruker 300 MHz spectrometer was used to record one-dimensional ^1^H and ^13^C-NMR and distortionless enhancement by polarization transfer (DEPT) spectra and two-dimensional NMR experiments. Samples were dissolved in deuterated chloroform (CDCl_3_) and the chemical shifts were reported in ppm values relative to the internal standard tetramethylsilane (TMS). 

The molecular weight of the different sesquiterpene lactones was determined using Gas Chromatography-mass spectrometry (GC-MS) and HPLC-MS. GC-MS analysis was performed using a Hewlett-Packard 6890 gas chromatograph equipped with HP-5 capillary column (30 m long, 250 µm internal diameter, and 0.25 µm film thickness). Helium was used as a carrier at a flow rate of 1 mL/min. The column was heated from 35 to 290 °C with the maximum reached temperature being 350 °C and the injector set at 300 °C in a splitless mode. Results were recorded as percent of total peak areas. The mass spectrometer employed in the GC–MS analysis was a Hewlett-Packard 7972 series mass selective detector in the electron impact (EI) ionization mode (70 eV). HPLC-MS analyses were performed using an Agilent 1100 Series LC/MS system. The automatic thermostated column compartment is a C18 reversed-phase column (250 × 4.6 mm i.d.; 5 μm). The injection volume was 20 µL for all samples. The mobile phase consisted of acetonitrile (A) and water (B). The gradient elution profile was 5 min 10:90 (A:B), 5 min 20:80 (A:B), and 10 min of 30:70 (A:B). The flow rate was set to 1 mL/min and the wavelengths of the ultraviolet detector at 205, 210, 215, 220, and 254 nm at room temperature. The molecular weight was determined using ESIMS. Ultraviolet-visible (UV-Vis) spectra were measured in methanol using a Jasco V-570 UV/VIS/NIR spectrophotometer. 

### 3.5. Cell Culture

The following human cancer cell lines: HCT-116, Jurkat, and HL-60 were purchased from the American Tissue Culture Collection (ATCC), East Rutherford, NJ, USA. Human squamous cell carcinoma HaCaT-II4 cells were kindly provided by Dr. Petra Boukamp (Heidelberg, Germany). The HaCaT-II4 cell line was cultured in DMEM containing 10% heat-inactivated FBS, 1% sodium pyruvate, 1% penicillin-streptomycin and 0.2% kanamycin antibiotics with normal calcium concentration in the medium (2 mM). The HCT-116, HL-60, and Jurkat cells were cultured in RPMI-1640 containing 10% heat-inactivated FBS, 1% sodium pyruvate, 1% penicillin-streptomycin and 0.2% kanamycin. Cells were grown at 37 °C, 95% air, and 5% CO_2_. Adherent cells were seeded into 96-well plates at a density of 1 × 10^5^ cells per well. When cells became 40%–50% confluent, they were treated with ethanol vehicle solvent (never exceeding 0.1%), as control, and with different concentrations of the tested *Achillea falcata* extracts and purified sesquiterpene lactones. HL-60 and Jurkat cells were treated in the same manner at a density of 2 × 10^5^ cells per well. The controls contained 0.1% ethanol in fresh culture media, which was equal to the highest ethanol concentration used in treated cells. These ethanol concentrations had no effect on the viability of all tested cell lines (data not shown).

### 3.6. Cytotoxicity and Cell Growth Assays

Drug cytotoxicity was assayed after 6 h of treatment using CytoTox 96^®^ assay (Promega Corp., Madison, WI, USA). Triton-X 100 with concentrations up to 1% of cell culture media was used as a positive control in this assay which measures the activity of the stable cytosolic enzyme LDH. Upon cell lysis, LDH is released and is measured in culture supernatants with a coupled enzymatic assay which results in the conversion of tetrazolium salt into a red formazan product. The absorbance of the latter is recorded at 490 nm using ELISA microplate reader. The respective cytotoxicities of the drugs were derived from the mean of triplicate measurements per condition (± SE) and normalized relative to the maximum toxicity of a Lysis Buffer. Cell growth at 24 h was assayed using the CellTiter 96^®^ non-radioactive cell proliferation assay kit (Roche Diagnostics, Basel, Switzerland). This assay is an MTT-based method which measures the ability of metabolically active cells to convert tetrazolium salt into a blue formazan product. The absorbance of this product was recorded at 595 nm using an ELISA microplate reader. Viability results were expressed as percentage of control and were derived from the mean of triplicate measurements per condition ± SE. The results of both cytotoxicity and cell growth assays were reproduced in at least three independent experiments.

### 3.7. Statistical Analysis

SPSS Version 18.0 and Microsoft Office Excel 2010 were used to calculate the best-fit regression model, the IC_50_ values, the mean standard errors, and the Independent Sample t-test and ANOVA with its associated post-hoc tests. The model regression functions for I.2 extracts against HCT-116, HaCaT-II4, HL-60, and Jurkat cells were best estimated as “logarithmic”. The model regression functions for the newly isolated compounds were best estimated as “logarithmic”, “growth”, and “exponential”. Statistical significance is claimed when the *p*-value is less than 0.05.

## 4. Conclusions

*Achillea falcata* was shown to be a rich source of sesquiterpene lactones. The anti-proliferative effect of six sesquiterpene lactones on HCT-116 cells was investigated. The biological activities of these compounds were related to the basic skeleton of the molecule, as well as to the presence and the positions of epoxides and hydroxyl functional groups.
